# Correction: Synthesis of acidic–basic Y zeolite from kaolin: catalytic and mechanism study in the cracking of polyethylene

**DOI:** 10.1039/d5ra90080h

**Published:** 2025-06-16

**Authors:** Samira Motamednejad, Li Gao, Kourosh Tabar Heydar, Reza Panahi, Bingsen Zhang, Mozaffar Shakeri

**Affiliations:** a Laboratory of Heterogeneous Catalysis, Chemistry and Chemical Engineering Research Center of Iran P.O. Box 14977-16363 Tehran Iran m.shakeri@ccerci.ac.ir +98-21-44787720; b Shenyang National Laboratory for Materials Science, Institute of Metal Research, Chinese Academy of Sciences Shenyang 110016 Liaoning China

## Abstract

Correction for ‘Synthesis of acidic–basic Y zeolite from kaolin: catalytic and mechanism study in the cracking of polyethylene’ by Samira Motamednejad *et al.*, *RSC Adv.*, 2025, **15**, 4861–4873, https://doi.org/10.1039/D4RA08978B.

The authors regret that the name of one of the authors (Samira Motamednejad) was shown incorrectly in the original article. The corrected author list is as shown here.

The Royal Society of Chemistry apologises for these errors and any consequent inconvenience to authors and readers.

